# Pan-cancer analysis identifies the oncogenic role of *CCNE1* in human cancers

**DOI:** 10.18632/aging.206163

**Published:** 2024-11-25

**Authors:** Yujie Ouyang, Ziyi Wu, Dilihumaer Aili, Chunhua Yang, Hui Zhang, Tong Wu

**Affiliations:** 1 Department of Dermatology, The Third Xiangya Hospital of Central South University, Changsha, Hunan 410013, China; 2 Department of Orthopedics, The Second Xiangya Hospital of Central South University, Changsha, Hunan 410011, China; 3 Department of Orthopedic Surgery, Affiliated Hospital of Traditional Chinese Medicine, Xinjiang Medical University, Ürümqi 830054, China; 4 Department of Orthopedics, Changsha Hospital, Xiangya Medical College, Central South University, Changsha 410005, China; 5 Department of Geriatrics, The Second Xiangya Hospital of Central South University, Changsha, Hunan 410011, China

**Keywords:** *CCNE1*, pan-cancer, prognosis, immune infiltration

## Abstract

Objective: To investigate expression, prognosis, immune cell infiltration of C*yclin E1* (*CCNE1*) in cancer.

Methods: We used TIMER and GEPIA datasets to analyze the differential expression of *CCNE1* in multiple tumors. GEPIA and Kaplan-Meier plotter databases were utilized to observe the prognostic significance of *CCNE1* in cancer. TIMER and cBioPortal databases were adopted for the analysis regarding immune infiltration and mutation respectively.

Results: The results showed that *CCNE1* was highly expressed in multiple cancers including BLCA, BRCA, CHOL, COAD, ESCA, HNSC, KICH, KIRC, KIRP, LIHC, LUAD, LUSC, READ, STAD, THCA, UCEC (*P* < 0.001) and CESC (*P* < 0.01). High *CCNE1* expression was associated with a poor overall survival prognosis in several cancers, including ACC, BRCA, KIRC, KIRP, LGG, LIHC, LUAD and MESO. Additionally, *CCNE1* expression was correlated with the cancer-associated immune infiltration level in BRCA, COAD, LUSC, STAD and THYM.

Conclusions: *CCNE1* is expected to be a potential biomarker for tumor prognosis and immune infiltration in various cancers.

## INTRODUCTION

The mechanism of tumorigenesis is complex, and the same gene has different mechanisms of action in different tumors [1, 2]. Pan-cancer expression analysis can effectively evaluate the mechanism of action of the same gene in different tumors, the predictive value of prognosis and survival of cancer patients and the effect of gene action from the molecular mechanism. Meanwhile, it can better help researchers in different specialties to clarify the overall role of genes in tumorigenesis. GEO and TCGA databases have a large number of functional genome data settings, and mainly focus on the role of these genes in different tumors, which is helpful for our relevant pan-cancer analysis [3–5].

One of the primary goals of cancer research is not only to understand tumor biology but also to improve patient outcomes through therapeutic interventions. Immunotherapies, including neoadjuvant and adjuvant treatments, represent a significant advancement in cancer therapy [6, 7]. However, the efficacy of these treatments often depends on the specific molecular and genetic landscape of the tumor. Understanding the expression of key genes, such as those involved in immune response or cell cycle regulation, across different cancers could help identify patients who would benefit most from immunotherapy. Pan-cancer analysis, by revealing gene expression patterns and immune infiltration across cancer types, plays a crucial role in informing the use of these therapies [8].

The *CCNE1* gene undergoes a series of processes such as replication, transcription, and translation to form *CCNE1* protein *in vivo*. The protein can act as a regulatory sub-unit of cyclin-dependent kinases and is important for maintaining genome integrity and the coordination of cell cycle processes [9]. Since the over-expression of the gene was found to be associated with cancer in 1990, a series of studies have shown that the protein encoded by the transcription of the gene can play different roles in multiple phases of the cell cycle [10–13]. Given its importance, *CCNE1* was selected as the focus of this study to better understand its function in cancer biology. In subsequent studies, various cancer types have been confirmed to be closely related to *CCNE1* over-expression, and the possible mechanism of action has also been analyzed [14–17].

In this study, we performed a pan-cancer analysis for the association of *CCNE1* expression with clinicopathological features of cancer. Moreover, several databases including TCGA, GEPIA, HPA, TIMER and STRING databases were applied to explore the association of* CCNE1* with prognosis, immune infiltration, and genetic mutation of tumor. This study’s innovative approach of integrating multiple datasets provides new insights into the potential clinical applications of *CCNE1* as a prognostic biomarker and its role in immune modulation across different cancers.

## MATERIALS AND METHODS

### Expression of *CCNE1* in pan-cancer

TIMER2 (tumor immune estimation resource) is an integrated gene data platform that can consistently analyze gene expression differences. Additionally, TIMER2 is widely used in cancer immunology research for its ability to estimate the abundance of six major immune cell types (such as CD4+ T cells, CD8+ T cells, and macrophages) using RNA-seq expression data from The Cancer Genome Atlas (TCGA). Its versatility and ease of use make it a powerful platform for studying the tumor immune microenvironment and its role in cancer progression and therapy responses [18]. TIMER2 was selected because of its wide application in gene expression differences and tumor immunology research, particularly for its reliability in estimating the gene expression differences and immune microenvironment, which was critical for our analysis. *CCNE1* was input into “GENE_DE” section that can evaluate the expression of *CCNE1* in different types, in the TCGA project. Statistical analysis of the association was performed via the test of purity-adjusted partial Spearman’s correlation. Results are shown via heat-maps and scatter plots. GEPIA2 (gene expression profiling interactive analysis) is an interactive web server to analyze expression of RNA which is derived from the Genotype Tissue Expression (GTEx) program and TCGA [19].

In our research, we obtained *CCNE1* expression by using this dataset and analyzed them via GEPIA2’s “Expression Analysis-Box Plots” section, with the DESeq2 package used for normalization and differential expression analysis. Specifically, the parameters were set with a log2FC cutoff of 1 and an adjusted *p*-value threshold of 0.01. In addition, the limma package was used for differential gene expression analysis in paired tumor and normal tissue comparisons. The limma package is applied to log-transformed data, where it uses empirical Bayes moderation to calculate moderated t-statistics for differential expression. We followed the platform’s default settings for the linear model fitting and moderated t-statistic computation.

### Association of *CCNE1* with overall survival and disease-free survival in cancer

Overall survival (OS) and disease-free survival (DFS) for all tumors in TCGA were obtained using the “Survival Map” module of GEPIA2. We set high (50%) and low (50%) cut-off to divide high and low cohorts of gene expression. Survival data were visualized with log-rank *P*-values, 95% confidence intervals, and hazard ratios. The expression of *CCNE1* in TCGA was obtained using the GEPIA2 “Pathological Stage Plot” program. Using “similarity for gene detection” module of GEPIA2, the top 100 targeted genes which are associated with *CCNE1* can be identified.

*CCNE1* expression levels were stratified into high and low expression groups. The stratification was based on the median expression value of *CCNE1* across the entire dataset. Samples with *CCNE1* expression above the median were classified as the high expression group, while those below the median were classified as the low expression group. GEPIA performs survival analysis based on gene expression levels. GEPIA uses log-rank test, sometimes called the Mantel-Cox test, for the hypothesis evaluation. The cox proportional hazard ratio and the 95% confidence interval information can also be included in the survival plot.

### Association of *CCNE1* with relapse-free survival in cancer

The Kaplan-Meier plotter that can evaluate genetic impact on prognosis of different cancers was used in our study. The relationship between the *CCNE1* gene and progression-free survival (PFS), OS, and first progression in different cancers from TCGA and GEO databases was analyzed using the Kaplan-Meier plotter. Setting “autoselect best cut-off”, the breast, ovary, lung, stomach and liver of the five tumors were divided into two groups. A log *p*-value of < 0.05 was considered statistically significant.

### Expression of *CCNE1* protein in cancer

UALCAN provided the protein analysis option through the TCGA and CPTAC datasets [20]. The CPTAC program of UALCAN can be used to obtain the *CCNE1* protein expression in different cancers.

### Analysis of *CCNE1* gene alteration and mutation

C-BioPortal is a complex site, which can visualize, and analyze the cancer genomic [21]. The frequency of *CCNE1* gene alteration, mutation type and alteration of copy number in tumors can be seen through “Cancer Types Summary” section of the cBioPortal in TCGA. Setting “TCGA Pan Cancer Atlas Study”, we can query the genetic alteration signature of the CCNE 1 gene via the “Quick select” section. Additionally, we used the “Comparison” section, analyzed data on the relationship among *CCNE1* genetic alterations, OS, PFS, and DFS. The results are shown as log-rank *P*-values.

### Immune infiltration analysis of *CCNE1* in cancer

Connection between *CCNE1* and immune infiltration of cancer was explored using TIMER database. “GENE_CORR” section can be used to analyze the association between gene expression and immune infiltration. Statistical analysis of the association was performed via the test of purity-adjusted partial Spearman’s correlation. Results are shown via heat-maps and scatter plots.

### Enrichment analysis of *CCNE1* associated genes

STRING can predict protein-protein interactions [22]. The top 50 lists of *CCNE1* binding-proteins, which set the required minimum interaction score to “low confidence”, the meaning of the network edge to “evidence”, and display as “no more than 50 interactors”, via STRING site. DAVID (database for annotation, visualization, and integrated discovery) is a functional annotation tool that investigates to clarify gene function [23]. The intersection analysis assesses *CCNE1* binding and interacting genes. The genes functional annotation chart was analyzed by the DAVID tool.

### Availability of data and materials

All data generated or analyzed during this study are included in this published article.

## RESULTS

### *CCNE1* gene expression

**Figure 1:**
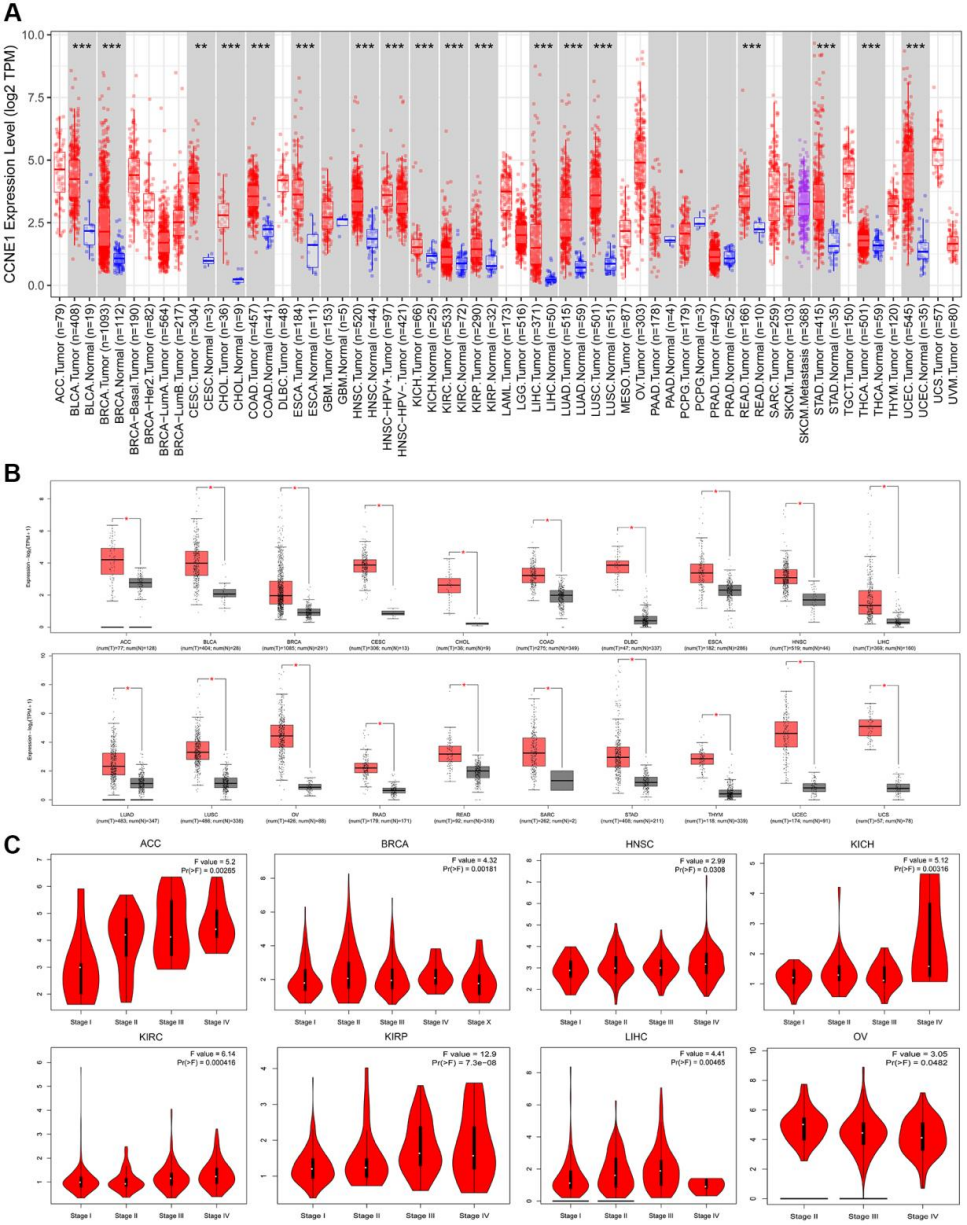
**Expression of *CCNE1* in different tumors and pathological stages.** (**A**) The expression of *CCNE1* in pan-cancer. The expression of *CCNE1* in tumor tissue is indicated in red, while the expression of *CCNE1* in normal tissue is shown in blue. (**B**) The expression status of *CCNE1* in normal tissue and cancer tissue. The expression of *CCNE1* in tumor tissue is indicated in red, while the expression of *CCNE1* in normal tissue is shown in grey. (**C**) Correlation of *CCNE1* with pathological stages in multiple cancers.

By comparing the expression of *CCNE1*, we investigated expression of *CCNE1* among cancer types in TCGA by TIMER2. *CCNE1* expression in the tumor tissues including BLCA, BRCA, CHOL, COAD, ESCA, HNSC, KICH, KIRC, KIRP, LIHC, LUAD, LUSC, READ, STAD, THCA, UCEC (*P* < 0.001) and CESC (*P* < 0.01) was higher than the corresponding normal tissues (Figure 1A). Analyzing the differences in our target gene expression, we used the GTEx dataset and used normal tissues as controls. *CCNE1* expression level was upregulated in ACC, BLCA, BRCA, CESC, CHOL, COAD, DLBC, ESCA, HNSC, LIHC, LUAD, LUSC, OV, PAAD, READ, SARC, STAD, THYM, UCEC and UCS, (*P* < 0.01, Figure 1B). We observed an association between *CCNE1* expression and tumor pathological stages (Figure 1C).

### *CCNE1* survival analysis

**Figure 2:**
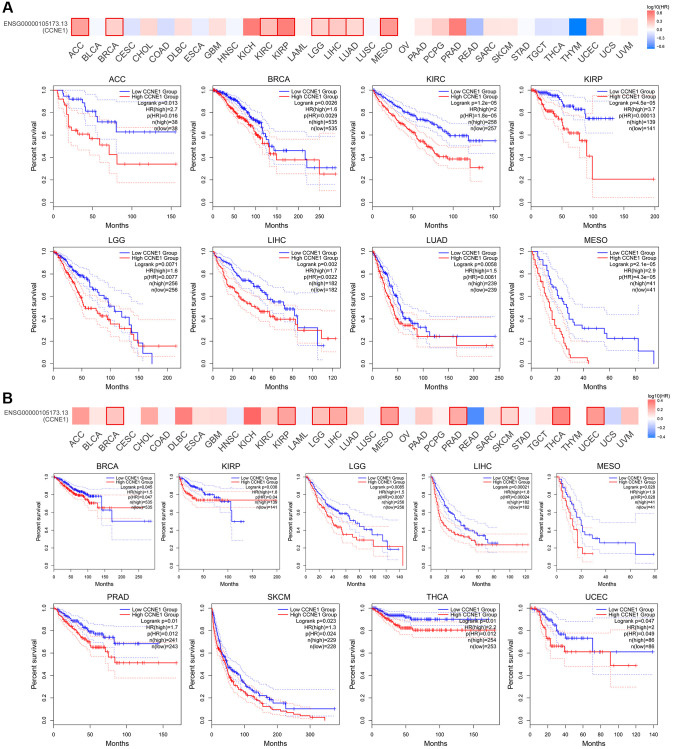
Connection of *CCNE1* with (**A**) overall survival and (**B**) disease-free survival in cancer. Tumors with higher *CCNE1* expression are indicated in red, while those with lower expression are shown in blue.

**Figure 3:**
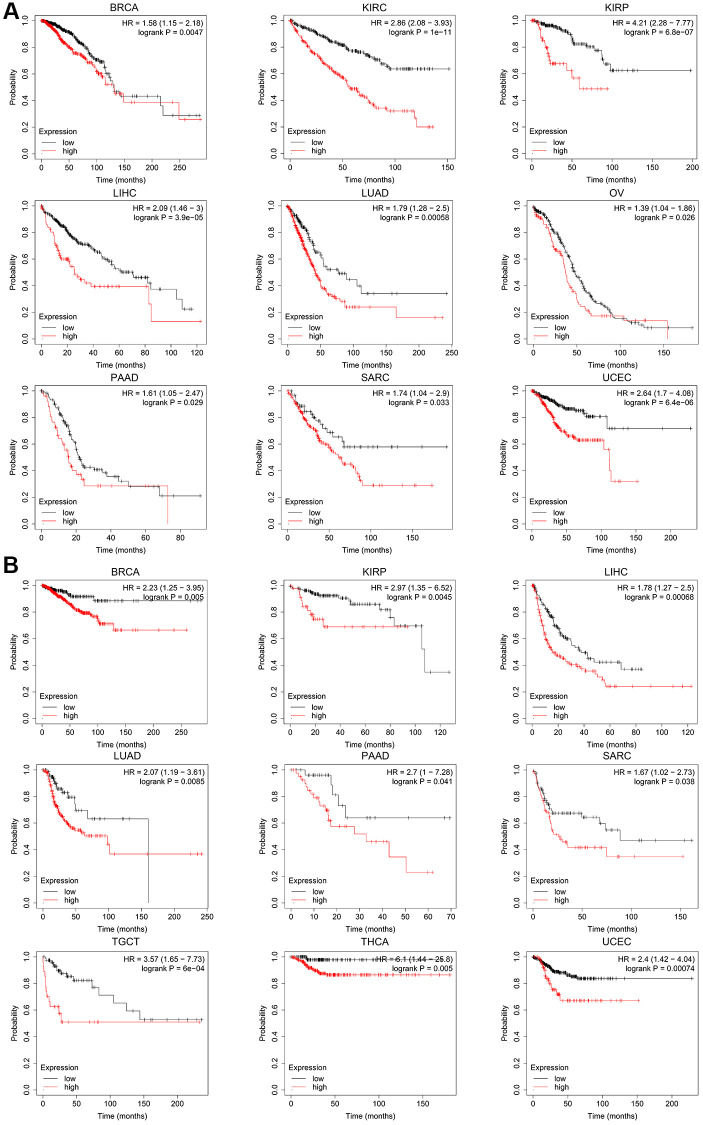
The Kaplan-Meier plotter reflecting *CCNE1* expression and (**A**) overall survival and (**B**) relapse-free survival. Tumors with higher *CCNE1* expression are indicated in red, while those with lower expression are shown in black.

According to the expression level of *CCNE1*, the tumor cases were divided into high and low groups. To investigate the correlation between *CCNE1* expression and tumor prognosis in different types of tumors, we used TCGA and GEPIA databases for analysis. High *CCNE1* gene expression was associated with poor prognosis of OS in cancers, including ACC, BRCA, KIRC, KIRP, LGG, LIHC, LUAD, MESO (Figure 2A). In addition, high gene performance of *CCNE1* showed a strong correlation with poor DFS in cancer types, such as BRCA, KIRP, LIHC, LGG, PRAD, MESO, SKCM, THCA and UCEC (Figure 2B). To explore the association between *CCNE1* and tumor prognosis, we performed further analysis via Kaplan-Meier plotters tool. High *CCNE1* gene expression was linked with poor OS in BRCA, KIRC, KIRP, LIHC, LUAD, OV, PAAD, SARC and UCEC (*P* < 0.01) (Figure 3A). The results of DFS were shown that a strong correlation between h highly expressed *CCNE1* and poor prognosis in BRCA, KIRP, LIHC, LUAD, PAAD, SARC, TGCT, THCA and UCEC (*P* < 0.01) (Figure 3B).

### *CCNE1* genetic change

**Figure 4:**
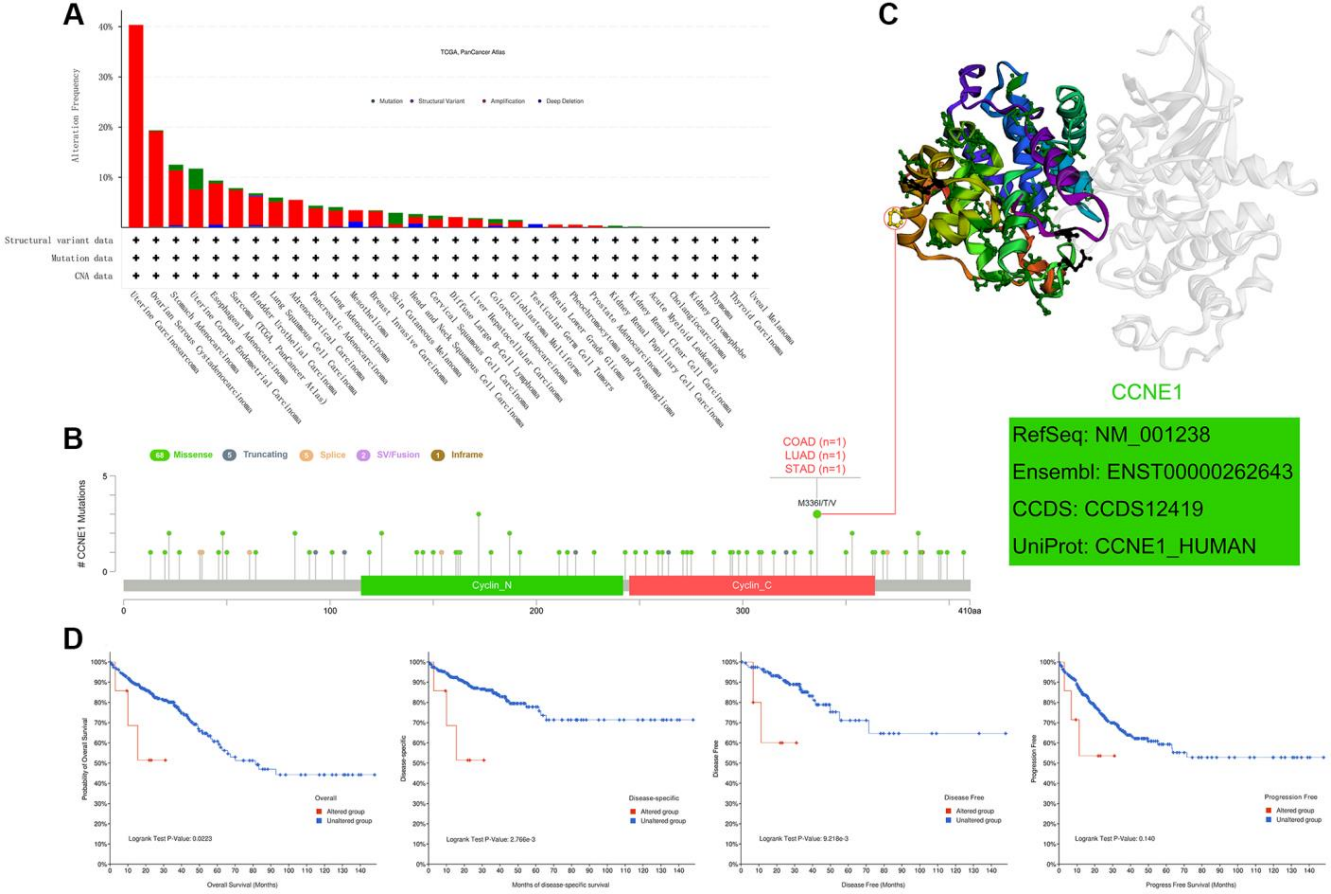
**Mutation features of *CCNE1* in cancer.** (**A**) Bar chart representing the distribution of different *CCNE1* mutation types across various cancers. (**B**) Lollipop plot highlighting the mutation sites of *CCNE1*, with higher frequency mutations displayed prominently. (**C**) 3D structural model of *CCNE1*, focusing on regions with the highest mutation frequency. (**D**) Kaplan-Meier curves linking *CCNE1* mutation status with survival outcomes (overall survival, disease-specific survival, disease-free survival, and progression-free survival) in COAD.

In this study, to analyze the genetic pattern of *CCNE1*, we used different tumor datasets from the TCGA cohort for our study. *CCNE1* has the highest genetic change frequency in uterine carcinoma patients (40%) (Figure 4A). And the main type is “amplification”. In UCEC, we found that the prominent types are the “amplification” and “mutation”, accounting for about 5% of the frequency of occurrence. As shown in the results, an interesting phenomenon is that most of the tumors listed in the figure are formed under the influence of *CCNE1*, and the “amplification”, is the predominant type. In Figure 4B, the analysis of the type, location and number of cases of *CCNE1* genetic changes is shown, including missense, truncating, and fusion mutations. Based on the analysis of our data, missense mutations were found to be the predominant type of genetic change in *CCNE1*, with M336I/T/V changes detected in 1 case of COAD, 1 case of LUAD, and 1 case of STAD (Figure 4B). The 3D structure of *CCNE1* protein is shown in Figure 4C. Potential association in different types of cancer was analyzed between *CCNE1* changes and prognosis by crude analysis of the change pattern. The clinical survival prognosis value of *CCNE1* alterations reflected prognosis in COAD patients with regard to OS (*P* < 0.05), DSS (*P* < 0.001), DFS (*P* < 0.001), but not PFS (*P* = 0.140) (Figure 4D).

### *CCNE1* protein expression

**Figure 5:**
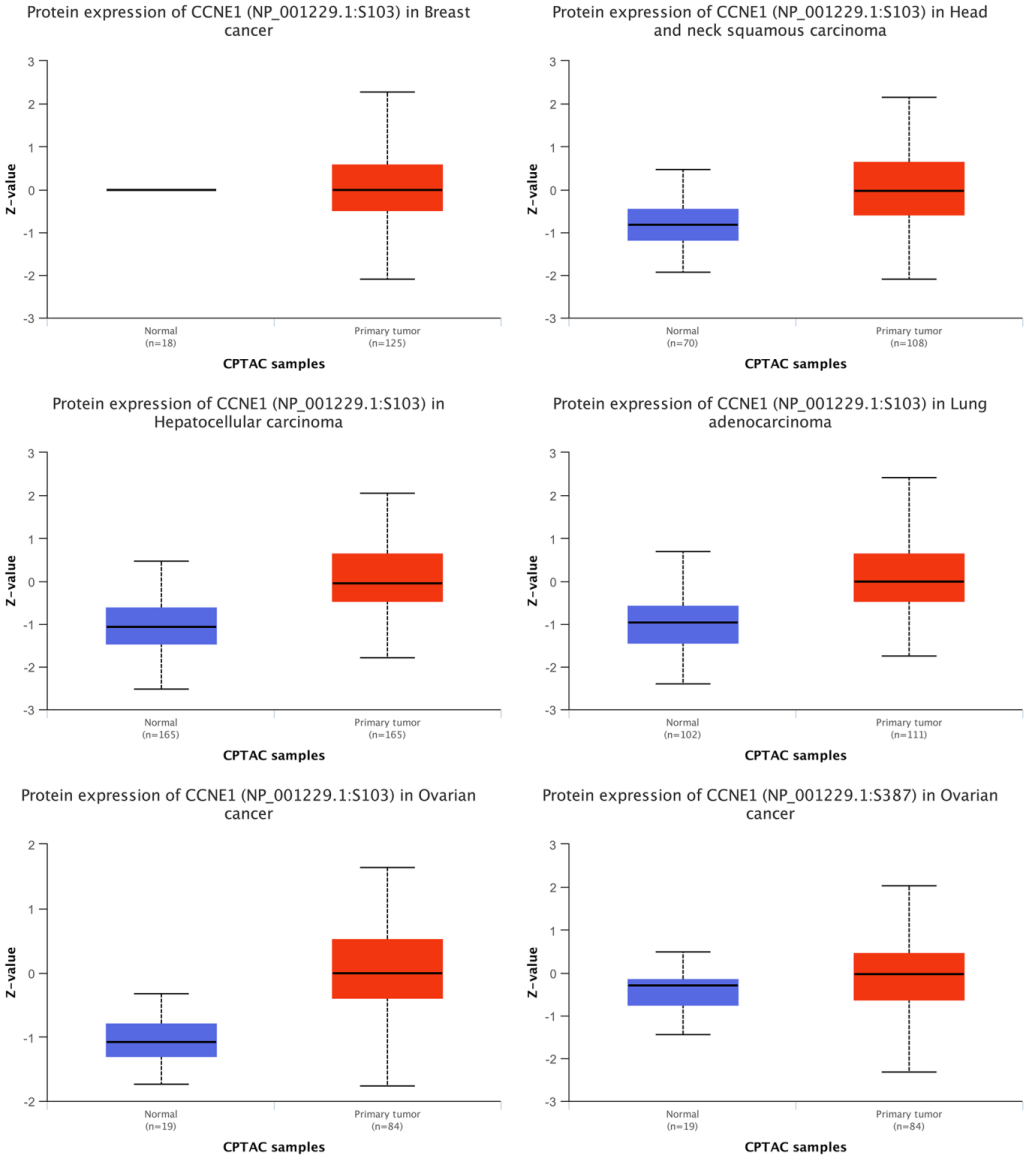
**Expression of *CCNE1* protein in multiple cancers.** The expression of *CCNE1* in tumor tissue is indicated in red, while the expression of *CCNE1* in normal tissue is shown in blue.

Through CPTAC, the expression of *CCNE1* protein in five types of tumors including BRCA, HNSC, HCC, LUAD, and OV were analyzed. We observed that the expression level of *CCNE1* protein was higher in BRCA, HNSC, HCC, LUAD and OV (*P* < 0.001) (Figure 5).

### Patients’ immune cell infiltration of *CCNE1*

**Figure 6:**
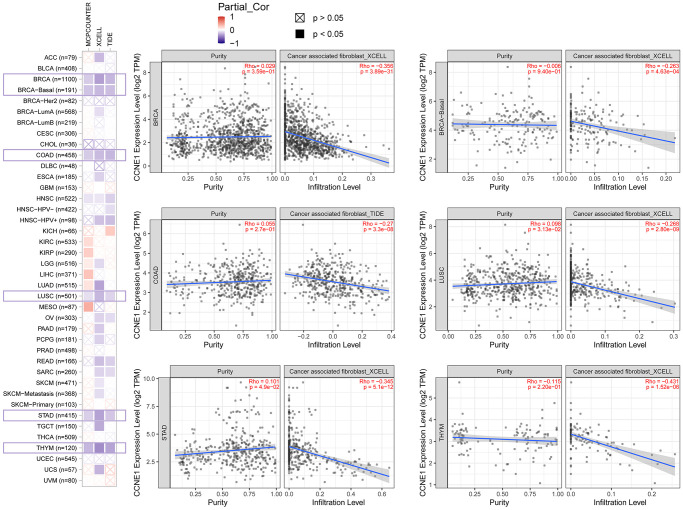
**Correlation between *CCNE1* expression and immune infiltration of cancer-associated fibroblasts in TCGA.** Scatter plot displaying the correlation between *CCNE1* mRNA levels and the infiltration levels of cancer-associated fibroblasts across TCGA cancer types. The strength of the correlation is represented by the Pearson correlation coefficient (r), and the p-values indicate the statistical significance of these associations.

To explore the involvement of *CCNE1* in immune infiltration and the role of this process in the initiation, progression and metastasis of tumor development, we used TIMER2, EPIC, MCPCOUNTER, CIBERSORT, CIBERSORT-ABS, QUANTISEQ, XCELL, naive_XCELL, central memory_XCELL, and effector memory_XCELL algorithms to analyze the correlation between immune cell infiltration and *CCNE1* differential expression in TCGA. *CCNE1* expression positively correlated with the cancer-associated immune infiltration level in BRCA, COAD, LUSC, STAD and THYM (Figure 6).

### *CCNE1* enrichment data analysis

**Figure 7:**
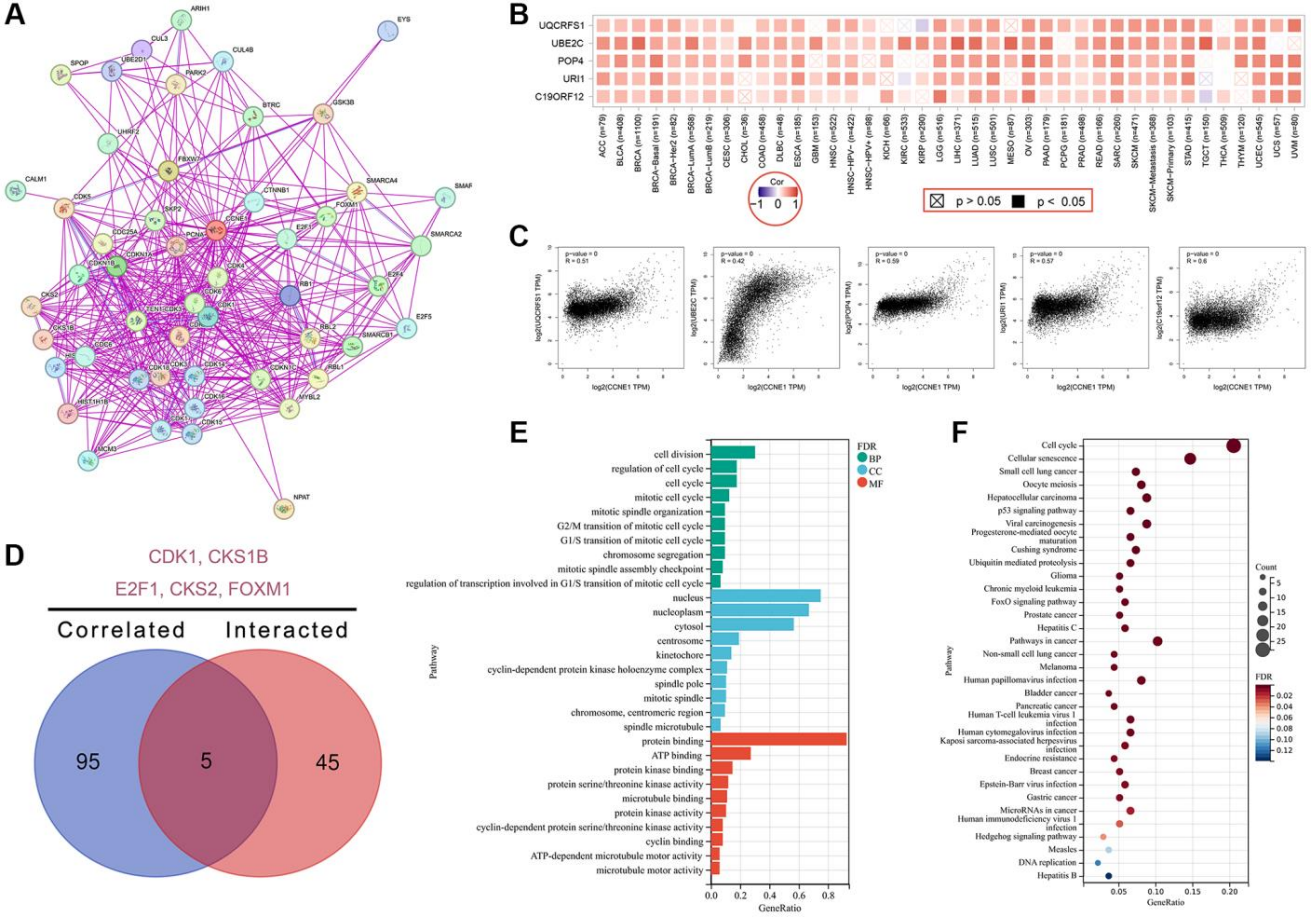
**Enrichment analysis for *CCNE1* interacted or related genes.** (**A**) Determining interacted genes of *CCNE1*. (**B**) Top 5 *CCNE1* associated genes in TCGA projects. (**C**) The corresponding heatmap map for correlation between *CCNE1* and top 5 related genes in various cancers. (**D**) An intersection analysis of *CCNE1* interacted and associated genes. (**E**) GO and (**F**) KEGG pathway analysis of *CCNE1* interacted or related genes.

In this study, the *CCNE1* gene molecule was investigated to screen for gene segments related to *CCNE1* binding protein and *CCNE1* performance, and a series of pathway enrichment studies were performed for this purpose, using the STRING online database. In our study, the network had 51 nodes which means genes and 397 edges that represent the links between binding genes (Figure 7A). We identified the top 100 genes that correlated with *CCNE1* expression, via GEPIA online databases. Additionally, the heatmap indicated a positive correlation between *CCNE1* top five related genes in cancer (Figure 7B) and the relationship between *CCNE1* and these five genes was also shown (Figure 7C). In Venn diagram, we analyzed the interaction and showed five common members including *CDK1*, CKS1B, E2F1, CKS2, and *FOXM1* (Figure 7D). Investigating functional and pathway enrichment analyses of *CCNE1*, we performed GO and KEGG enrichment analyses via the DAVID 6.8 online tool. GO enrichment analysis data suggested that most of genes are linked to regulation of cell division in BP category, nucleus in CC category, and protein binding in MF category (Figure 7E). KEGG analysis indicated that the *CCNE1* and correlated genes and interacted genes were mainly enriched in cell cycle and cellular senescence pathway (Figure 7F).

## DISCUSSION

The main role of CCNE 1 encoding cyclin E1 is to promote the transition of the cell cycle from G1 to S phase, thereby facilitating the initiation of DNA synthesis [24]. More and more studies on the role of *CCNE1* in tumor tissues have been reported, for example, *CCNE1* over-expression in BRCA [25], BLCA [26] and OV [27] tissues are associated with poor tumor survival, although there are also some opposite results [28–30]. Previous studies have reported that 19q12 amplification mutations in *CCNE1* are associated with tumor types, especially in uterine tumors, high-grade serous ovarian cancer, and gastroesophageal cancer [31–35]. Moreover, the findings from relevant scholars suggest that such changes enhance genomic instability, genome-wide proliferation, and resistance to cytotoxicity [31–35]. However, whether there is a corresponding common pathway of *CCNE1* in its translation, transcription, expression and other processes to produce effects in different tumor pathogenesis is still not clear. After an extensive literature review, there is no unified conclusion on the results of our pan-cancer analysis of *CCNE1* [36, 37]. But interestingly, we found that the conserved structure of *CCNE1* protein exists in different types of tumor tissues [25, 38, 39], which also implies that *CCNE1* may have a similar mechanism in the action of *CCNE1* in tumor tissues. Therefore, the CPTAC, GEO, TCGA databases were analyzed in our study to examine the role of *CCNE1* in 33 tumors in terms of gene mutation, gene expression and molecular characterization of *CCNE1*.

Our results suggested that the abnormal expression of *CCNE1* occurs frequently in multiple types of tumors. We found that *CCNE1* expression level in the tumor tissues of BLCA, BRCA, CHOL, COAD, ESCA, HNSC, KICH, KIRC, KIRP, LIHC, LUAD, READ, STAD, THCA, UCEC, and CESC was higher than the corresponding normal tissues. Functionally, *CCNE1* plays a crucial role in driving the transition from G1 to S phase in the cell cycle by forming a complex with CDK2, thereby promoting DNA replication and cell division. This process is critical in maintaining normal cellular homeostasis. However, in cancer, aberrant *CCNE1* expression can lead to uncontrolled proliferation [40]. For instance, the overexpression of *CCNE1* in ovarian cancer has been shown to promote genomic instability through its interaction with CDK2, which accelerates the cell cycle and impairs DNA repair mechanisms. *CCNE1* has been implicated in epithelial-mesenchymal transition (EMT), further promoting cancer cell dissemination [41]. These findings underline the multifaceted role of *CCNE1* not only in cell cycle regulation but also in driving aggressive cancer phenotypes through various molecular pathways. From the above results, it is not difficult to conclude that the down-regulation of *CCNE1* expression will become the next direction to focus on. Our study found that the abnormal expression of *CCNE1* occurred not only in one tumor, but also in a variety of tumor types. For example, in the tumor tissues of BLCA, CESC, and ESCA, *CCNE1* expression levels are higher than normal tissues. Furthermore, in the tumor tissue of HNSC, KIRP, and THCA, the results were the same as above. The elevated expression of *CCNE1* in various cancers indicates its significant role in tumorigenesis and cancer progression [42]. Clinically, high *CCNE1* expression has been correlated with several critical aspects of cancer behavior and patient outcomes. Elevated *CCNE1* levels are often associated with increased tumor aggressiveness and invasiveness. This is likely due to its role in driving the cell cycle progression from G1 to S phase, promoting rapid cell division and proliferation. Studies have shown that higher *CCNE1* expression can lead to enhanced metastatic potential, making it a marker for aggressive cancer phenotypes. *CCNE1* overexpression may influence the response to various cancer treatments. For instance, cancers with high *CCNE1* expression may exhibit resistance to certain chemotherapeutic agents that target cell cycle pathways. This resistance can be attributed to the rapid cell cycle progression driven by *CCNE1*, which might reduce the efficacy of treatments designed to halt cell division. On the other hand, targeting *CCNE1* directly or its associated pathways could offer a novel therapeutic approach, potentially improving treatment outcomes for patients with *CCNE1*-overexpressing tumors. As demonstrated in our study, high *CCNE1* expression is associated with poor overall survival in several cancers, including ACC, BRCA, KIRC, KIRP, LGG, LIHC, LUAD, and MESO. This highlights its potential as a prognostic biomarker. Patients with elevated *CCNE1* levels may require more aggressive and tailored therapeutic strategies to improve their prognosis. Furthermore, innovative therapeutic approaches focusing on the downregulation of *CCNE1* have shown promise in mitigating tumor growth and enhancing treatment efficacy [43].

Among these, RNA interference (RNAi) technologies and small molecule inhibitors specifically targeting *CCNE1* activity have emerged as effective strategies [44]. For instance, RNAi approaches, such as siRNA and shRNA, have been shown to successfully silence *CCNE1* expression, reducing tumor growth in breast cancer [45]. In addition to RNAi, small molecule inhibitors like Adavosertib, a WEE1 inhibitor, have been explored for their ability to inhibit cyclin E/CDK2 complexes, slowing down cell cycle progression in cancers with *CCNE1* amplification [46]. These advancements indicate that targeting *CCNE1* pathways can offer substantial therapeutic benefits, particularly for patients with *CCNE1*-overexpressing tumors where traditional treatments have failed [47].

Despite the high level of CCNE 1 expression in many tumors, different conclusions from previous studies. To explore the association of CCNE 1 with different tumor outcomes, a meta-analysis study found that over expression of CCNE 1 was associated with poor survival in cancer patients [30]. In this research, in this study, there was a statistically significant relationship between a high expression of CCNE 1 and a poor survival prognosis (*P* < 0.01). The reason may be different data processing methods and new survival information. At the same time, we explored the relationship between OV pathological stage and *CCNE1* expression level through the “Pathological Stage Map” module of GEPIA2 (*P* < 0.01). In addition, we performed a survival analysis of OV by the Kaplan-Meier plotter approach. The results suggested that *CCNE1* expression was not statistically significant with the clinical prognosis of DFS and OS. Therefore, the current evidence based on clinical big data can only prove that the over expression of *CCNE1* is correlated with OV, and cannot support the clinical prognosis role of OV. Our study also found that the poor prognosis of different tumors was closely associated with the high expression level of CCNE 1, suggesting that the high expression status of CCNE 1 may be a biomarker of clinical prognosis in cancer patients. Meanwhile, it also means that down-regulation of *CCNE1* expression may be one of the methods to improve the prognosis of patients. Recent studies have confirmed that PKMYT1 inhibitors may be an effective approach for the treatment of *CCNE1*-amplified cancers [34].

Tumor microenvironment (TME) is a setting that promotes tumor progression, and tumor cells often use this setting to escape the surveillance of immune function. In addition, TME also plays an important role in reflecting the therapeutic effect and predicting the clinical outcome of tumors. The research results of relevant scholars showed that TME components include tumor-associated mesenchymal stem cells, extracellular matrix, lymphocytes, fibroblasts [48–51]. Tumor immune-infiltrating cells play an important role in immune regulation by transferring from blood to tumor tissue [52]. *CCNE1*, as a major component of focal adhesions, plays a vital role in the extracellular matrix [53]. Importantly, CCNE 1 may also have significant effects on the immune response and TME. In our research, we analyzed the relationship between immune cell infiltration and differential expression of *CCNE1* using CIBERSORT-ABS, XCELL, MCPCOUNTER, QUANTISEQ, EPIC, CIBERSORT central memory_XCELL, effector memory_XCELL algorithms, and TIMER2. In different tumors, high expression of *CCNE1* is associated with cancer-associated fibroblasts, especially in BRCA, COAD, LUSC, STAD, THYM. It has been reported that cancer-associated fibroblasts and endothelial cells have been able to exert tumorigenic effects in the TME by secreted various growth factors, cytokines and chemokines and promoting the degradation of the extracellular matrix [54, 55]. However, in our study, there was no obvious association between *CCNE1* expression and MSCs, monocytes, or bone marrow-derived suppressor cells, but the link between *CCNE1* and TME through cancer-associated fibroblasts may explain the prognostic impact of *CCNE1* in different cancers. At the same time, we found no significant correlation between CCNE 1 expression and MSCs, monocytes, or bone marrow-derived suppressor cells. MSCs are regulators of the tumor niche and are involved in tumorigenesis and metastasis. *CCNE1* was previously reported to be important in the proliferation and colony formation of MSCs [56, 57]. Our study reveals a significant correlation between *CCNE1* expression and increased immune cell infiltration in multiple cancers, including breast, colon, lung, stomach, and thymic cancers. By employing various algorithms, we’ve confirmed that elevated *CCNE1* levels enhance immune presence, aligning with its known roles in cell cycle regulation and tumorigenesis. Notably, this correlation suggests *CCNE1*’s involvement in modulating the tumor microenvironment, which could influence immune cell recruitment and activation, thus presenting potential therapeutic implications for enhancing cancer immunotherapy effectiveness. More analyses on immunotherapy to further reveal the biological properties and roles of *CCNE1* in different tumours are needed to provide an important reference for subsequent studies.

Moreover, genetic mutations also play a critical role in the mechanism of action of carcinogenesis [58]. The overexpression of *CCNE1* has been found in a variety of tumors, so, we further explored the corresponding mutation characteristics of *CCNE1*. Kang et al. showed that CCNE 1 gene amplification leads to poor prognosis in cancer patients [59]. *CCNE1* gene mutations include amplification, missense mutations, truncating mutations, and fusion mutations. Missense mutations such as M336I/T/V result in amino acid substitutions that may impact the stability and function of the *CCNE1* protein. Amplification may lead to overexpression of *CCNE1* protein, causing dysregulation of cell cycle control and promoting tumorigenesis. Fusion mutations can produce fusion proteins with novel functions or loss of original functions, affecting cell proliferation and differentiation. Truncating mutations may result in the production of truncated proteins lacking essential functional domains, thereby impairing their normal function. *CCNE1* plays a critical role in cell cycle regulation, and its mutations and overexpression are closely associated with the development of various cancers. *CCNE1* mutations may lead to cell cycle dysregulation, promoting cancer cell proliferation. A detailed analysis of different mutation types can enhance our understanding of the role of *CCNE1* in cancer development and progression, providing a theoretical basis for precision medicine.

Our study showed that CCNE 1 had the highest frequency of changes in uterine cancer patients, at 40%, whose main type is “amplification”. And we also found that in UCEC, the more prominent types are the “amplification” and “mutation”, accounting for about 5% of the frequency of occurrence. Above all, most of the tumors listed in the study are formed under the influence of *CCNE1*, and the “amplification” is the predominant type. In conclusion, *CCNE1* amplification may effectively predict the occurrence of adverse prognostic events in cancer patients, and the control of *CCNE1* amplification will become a hot research direction for the control of adverse prognostic events. This suggested that the enriched pathways associated with CCNE 1 could be used as potential therapeutic options for the treatment of cancer patients. Additionally, the analysis found that the amplification of *CCNE1* was associated with poor prognosis of patients, so the *CCNE1* gene mutation may also be a potential molecular marker for poor prognosis in endometrioma patients.

For the first time, the top 100 proteins were screened by software and finally found *CDK1*, CKS1B, E2F1, CKS2, and *FOXM1* to be associated with *CCNE1*. Although the different roles of the above proteins in tumor pathogenesis have been reported, little is known about the exact pathogenic mechanism of tumors, which is also the focus of our future studies. Meanwhile, in this study, the CPTAC dataset was used to explore the expression of *CCNE1* protein in breast cancer, hepatocellular carcinoma, head and neck squamous cell carcinoma, ovarian cancer, and lung adenocarcinoma.

We used GO enrichment analysis and KEGG pathway enrichment analysis to investigate the differential expression signatures of CCNE 1. The results found that the differentially expressed CCNE 1 was mainly associated with the regulation of cell division in the BP class. Moreover, it is also related with the protein binding regulation of MF class and the nuclear regulation of CC class. Importantly, miR-30c-2-3p has an important role in cancer cell migration, by regulating CCNE 1 gene expression to regulating cytokine expression, invasion and cell proliferation in breast cancer cells. Previous study has shown that miR-30c-2-3p negatively regulates NF-κB signaling and cell cycle progression by downregulating CCNE 1 in breast cancer, resulting in reduced cytokine expression *in vitro*, cell invasion, and cell proliferation *in vitro* [60]. In addition, previous studies in gastric cancer showed that CircDENND2A can promote the progression of non-small-cell lung cancer by regulating the *CCNE1* signaling pathway [61]. However, there were some limitations existing in our study. The data used for analysis were obtained from online services. More cell-based studies and clinical experiments are needed to confirm our findings and to further explore interactions between relevant molecules, the precise mechanisms involved, and the potential clinical applications of *CCNE1* in cancer.

## CONCLUSIONS

In this study, a series of pan-cancer analyses were performed to determine the relevance of *CCNE1* in pan-cancer and its potential predictive value. We found that the expression of *CCNE1* is related to clinical prognosis, immune infiltration of tumor cells, and gene mutation. The pan-cancer analysis of *CCNE1* can analyze the role and effect of CCNE 1 in tumor development and development from multiple perspectives, and can provide some help for clinical diagnosis, treatment and basic research.
